# Common Ground in Demonstrative Reference: The Case of Mano (Mande)

**DOI:** 10.3389/fpsyg.2020.543549

**Published:** 2020-12-17

**Authors:** Maria Khachaturyan

**Affiliations:** Helsinki University Humanities Programme, University of Helsinki, Helsinki, Finland

**Keywords:** deixis, common ground, reference, ethnography, interaction, interactional history, corpus, Mande languages

## Abstract

That demonstratives often have endophoric functions marking referents outside the physical space of interaction but accessible through cognition, especially memory, is well-known. These functions are often classified as independent from exophoric ones and are typically seen as secondary with respect to spatial deixis. However, data from multiple languages show that cognitive access to referents functions alongside of perceptual access, including vision. Cognitive access is enabled by prior interactions and prior familiarity with the referents. As a result of such interactions, the interlocutors share a great deal of knowledge about the referents, which facilitates reference to objects in the interactive field. The centrality of common ground in reference to an object at the interactive scene challenges the often assumed classification of demonstrative reference into exophoric and endophoric. I illustrate this idea throughout the paper by using first-hand data from Mano, a Mande language of Guinea. Adding another argument in favor of viewing demonstrative reference as a social, interactive process, the Mano data push the idea of salience of non-spatial parameters further and emphasizes the importance of short and long-term interactional history and cultural knowledge both for the choice of demonstratives in exophoric reference and for the structuring of the demonstrative paradigm.

## Introduction

It is generally accepted that the exophoric reference to objects in the physical space of interaction is more basic than the endophoric reference used to track referents in discourse or in reference to discourse itself (Diessel, 1999; Levinson et al., 2018). Much scholarly attention has been directed to the exophoric use using a targeted elicitation methodology (i.e., Coventry et al., 2014; Wilkins, 2018). In such studies, interaction participants are only given the very lean and abstract characteristics of “speaker” and “addressee.” Yet such an approach obscures the fact that participants in real-life speech events come to any particular interaction with a set of expectations and background knowledge. This fact challenges the standard classification of demonstrative functions that contrasts exophoric and endophoric uses (on that point, see also Agha, 1996)^1^. Indeed, a referent physically present at the interactive scene often belongs both to the deictic sphere of interaction and to the non-deictic sphere of common ground which includes the participants’ mutual knowledge, beliefs and suppositions (Clark et al., 1983). As argued by Coventry et al. (2014, p. 49), “the perception of space is not constrained solely by the characteristics of the physical environment, but is mediated by high-level knowledge about the objects being perceived.” In this paper, by using first-hand, to a large extent previously unpublished data from Mano (Mande, Guinea), I extend this important conclusion by arguing that such mixed endophoric-exophoric uses cannot be accounted for unless one sees interactants as social actors and interaction as loaded with history (Hanks, 1990, 2005).

Let us compare two examples from Mano. In (1) the speaker is sitting side by side with the addressee on a sofa. The addressee is reading a book, occasionally using sticky notes to markup pages. The package of sticky notes referred to in (1) by using the demonstrative 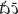 is placed between the interlocutors. It has, perhaps, never been seen before in the household in question (it is not named but is referred to by a 3sg pronoun) and has not been discussed previously. The interlocutors were not attending the referent in a joint activity: in fact, the addressee’s attention is focused on the book rather than on the sticky notes. For this reason the speaker makes a pointing gesture, which helps her secure the addressee’s visual attention to the referent with the first attempt.


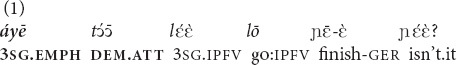


“This one is going to finish, right?” [Mano, own fieldnotes].

In (2) a young street vendor is addressing two persons passing by about 1.5 m away on a motorbike. The two motorbike riders are engaged in a conversation, so they were not attending the referent before it was mentioned. The vendor is suggesting they buy popcorn balls, a very popular snack found on many corners of Mano villages and towns, something the interlocutors are surely used to and expect to see. For this reason no visual attention is required. Moreover, precisely because she expects the addressees to be familiar with the referent (which is also marked with the possessive pronoun kà “your”) and because it is difficult to draw the addressees’ visual attention, the speaker chooses the 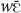 demonstrative.


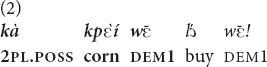


“Your popcorn (lit.: this corn of yours), buy it!” [Mano, own fieldnotes].

The choice between the demonstratives 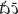 and 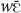 is not motivated by the differences in physical distance between the speaker and the object: in both cases the object is located within the arm’s reach (on a sofa next to the speaker or on a stand). The position in the interactional sequence also does not appear to matter: both (1) and (2) concern first mentions of referents in a given interaction. Instead, the choice is motivated by the degree of prior familiarity with and the existence of shared knowledge about the referent. The Mano exophoric demonstratives 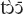 (and 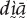) indicate that additional visual attention coordination is required in order to identify the referent and for this reason they are typically accompanied by deictic pointing, as in (1). In contrast, the demonstratives 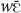 (and *yā*), which may be used exophorically, as in (2), or endophorically, do not encode such a requirement. Although they may be used for establishing joint visual attention, they are also (and even more frequently) used in situations where such visual attention coordination is already established or where it is impossible or unnecessary, when the speaker has reasons to believe that the referent is salient enough for the purposes at hand. In these situations, referent identification is primarily based on the interlocutors’ mutual knowledge of the referent, which is derived from prior interactions or broader cultural knowledge, as in (2).

## Interactional Perspective on Demonstrative Reference

Demonstratives are linguistic expressions serving “to coordinate the interlocutors’ joint focus of attention” on a reference object (Diessel, 2006, p. 464). The physical co-presence of interlocutors is understood as the prototypical property of interactional settings, and coordination of attention on objects in space is considered the primary function of demonstratives, primordial in phylogeny (Tomasello, 2008), best described in the literature (Levinson et al., 2018) and also seen as the source of further functional development and grammaticalization of non-spatial, endophoric functions (anaphora, discourse deixis, recognitional function, see Diessel, 1999). Even synchronically, anaphora is sometimes seen as a metaphoric extension of space (Anderson and Keenan, 1985).

A considerable volume of ethnographic and experimental research on demonstratives has shown that spatial distinctions cannot be reduced to mere physical distance. Natural, artificial, and culturally imposed boundaries (rivers, valleys, island boundaries; walls; family spaces, boundaries of the village) also contribute to the conceptualization of the proximity vs. distance contrast (as in *this village*, which can cover quite an extensive space, Margetts, 2018, see also Hanks, 1990; Enfield, 2003). In other words, in formulation and interpretation of demonstrative reference, the speakers engage with a great deal of knowledge about the social world and the space they inhabit is not merely physical but interactional. Thus, in Lao, the choice of demonstrative in a two-part system is determined by the position of the referent with respect to the interactionally defined here-space of the speaker (Enfield, 2003).

In addition to the knowledge about the space of interaction, in real-world interactional settings the referents themselves do not appear as timeless artifacts, as is often the case in experimental studies, but are loaded with history. On one level, the establishment of joint attention and referent identification are parts of an interactive process (Hindmarsh and Heath, 2000; Eriksson, 2009; Etelämäki, 2009). Several languages have been attested where demonstratives encode different stages in that process. Thus, in addition to the speaker-centric spatial distinction between a proximal and a distal demonstrative, another core semantic distinction in the Turkish demonstrative paradigm is whether the referent is in the addressee’s focus of attention (Küntay and Özyürek, 2006). Such cases concern the history of a given interaction within several interactional turns (see section “Common Ground and Interactional History”).

On a further level, as argued by Clark et al. (1983), reference resolution can be predicated on mutual knowledge built in long-term local histories of interaction, stretching beyond the interaction at hand. Thus, as the authors argue, the local history of interaction plays a role when a speaker points to a group of men and says “That is what George will look like very soon”: the reference is interpretable upon a condition that something has been said between the two interlocutors about George, e.g., that he is gaining or losing too much weight, and that one of the men in the group looks overweight or underweight. Thus, meaning and reference are established in time, as part of incremental building of common ground within and across interactional encounters (Deppermann, 2018; Harjunpää et al., 2021).

A further level of complexity arises when interaction participants are not seen as merely speaker and addressee engaged in interaction, but as cultural beings who, by virtue of their membership in a particular collectivity (community of practice, speech community), operate with a great deal of common knowledge. Such culturally shared knowledge, which is (presumably) available to most if not all members of a given collectivity, became apparent as a determiner in an experiment discussed in Clark et al. (1983), where the subjects were shown a photograph of President Reagan and David Stockman, then the director of the Office of Management and Budget. When asked, “You know who this man is, don’t you?” the overwhelming majority of subjects understood “this man” to be Reagan, not Stockman, who was much less known. Furthermore, interaction participants are also social actors engaged in specific partially scripted social activities. Such activities presuppose a specific optic through which some objects are seen and a specific way they are referred to Hindmarsh and Heath (2000). In particular, even when objects are referred to for the first time in a given interaction, they may be partially anticipated. For example, in Yucatec Maya, a shaman wrapping up a medicine for his patient may refer to it using a non-immediate deictic, despite the fact that the referent is immediately accessible, which would in other contexts warrant the use of an immediate deictic. This use, as Hanks (2005) explains, is in part because the referent, a medicine, is presented as mutually known and expected in the context of shamanic practice and such uses are typically covered by a non-immediate deictic.

A given referential act is thus part of different mutually constituting levels. On the one hand, it belongs to the level of the temporarily unfolding interactional process of reference resolution, which in turn is part of a longer-term interactional history involving the same communicating individuals. On the other hand, these individuals participate in communication not only as communicating agents, but also as social agents occupying different positions in social fields (Bourdieu, 1990) routinely dealing with and talking about specific kinds of artifacts. In other words, any given interaction is *embedded in* (Hanks, 2005), and is an instantiation of, a social field whereby the properties and the relationships between the positions in the social field are projected onto a very general structure of the interactional space. Thus, the properties and relationships in the triad shaman–patient–medicine is projected onto a given triad of speaker, addressee and referent and motivates the choice of the referential expression. Both kinds of embedding, embedding in interactional history and in social fields, transform referents in the interactive space from physical to social artifacts known to the interactants: while short- and long-term interactional history provides situated knowledge to given participants, the social field provides more general knowledge available to wider collectivities participating in the same social field.

The following discussion is organized in the following way. Section “Mano Demonstrative System” presents four Mano demonstratives as they are used in naturally occurring referential acts. Section “Demonstratives 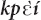 and 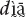
” offers some basic morphosyntactic information on the four Mano demonstratives. Section “Demonstratives 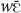 and yā” presents the functions of the demonstratives 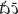 and 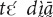 in more detail. Section “Demonstratives 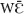 And yā” is dedicated to the demonstratives 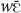 and *yā*, their endophoric (discourse reference, anaphora and recognitional function) and exophoric functions, as well as the contrasts between the two markers. Section Semantics and Pragmatics of Mano Demonstratives is an interim summary where I disentangle the semantic and pragmatic components of the meaning of Mano demonstratives. Section “Common Ground and Reference Resolution in Interaction” presents the demonstratives 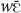 and *yā* in a broader interactional context which provides the interaction participants with knowledge about referents and motivates the use of 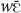 and *yā*. Section “Common Ground and Interactional History” illustrates the interactional process of referent identification and the role of idiosyncratic mutual knowledge built in local interactions, in particular, between friends and family members. Section “Common Ground and Social Fields” deals with knowledge proper to specific social fields, namely the domain of ritual practice (“traditional” and Christian) and specificities of demonstrative reference proper to these domains. I discuss the findings in section “Discussion” and make my conclusions in section “Conclusion.”

## Mano Demonstrative System

Mano (*mááwè*) is a Southern Mande language spoken by 305,000 people in Liberia (Ethnologue^2^) and, according to different estimations, by 66,000 (Guinean census performed in 2014, Bah and Bangoura, 2017) and 95,000 (Ethnologue) in Guinea. A grammar of Mano can be found in Khachaturyan (2015), and for a typological portrait of the language, see Khachaturyan (2020a). The demonstrative system is subject to dialectal variation; for a preliminary account, see Khachaturyan (2018a). Despite the widespread multilingualism, Mano is well-preserved and well-transmitted to children; (quasi)-monolingual repertoires have also been attested (Khachaturyan and Konoshenko, forthcoming).

Mano, just like other Mande languages, is a largely isolating language. It has a fixed S-Aux-O-V-X word order, where Aux is an auxiliary inflected for person and number and agreesing with the subject, and X is any post-verbal argument expressed by a postpositional phrase. With three tonal levels, it has quite a large number of tonal morphemes but a relatively small inventory of segmental morphemes. Thus, besides very minimal derivational morphology, the only two inflectional nominal forms are the low-tone construct form (CSTR), appearing on the head of noun phrases with specific kinds of preposed dependents, and a high-tone form (H) used with demonstratives. Definiteness is not grammaticalized in the language, and although certain grammatical markers take on the functions of marking definiteness, they are never obligatory (Khachaturyan, 2020b).

This paper is based on the data from the Central Guinean dialect of Mano, Maa (máá), drawn from a corpus of recordings of spontaneous speech collected by the author during more than 15 months of fieldwork. All the examples taken from the Mano oral corpus are marked with MOC. Some examples come from fieldnotes [fieldnotes]: these are utterances that I overheard and noted and then asked the consultant to comment on them and, if necessary, correct. A minor fraction of examples are elicited (el.). All elicited utterances were contextualized and discussed with the primary language consultant. Whenever applicable, square brackets [] indicate exact discourse context preceding or following the utterance in question, parenthesis () indicate a summary of the preceding or following context or provide other textual commentaries. No systematic questionnaire study of exophoric use has been conducted, which represents a major limitation of the present study. However, since the focus of the paper is the role of common ground in reference resolution, some of which is acquired in interaction between specific individuals (inhabitants of the same village, or family members), the observational data are adequate for the analysis.

The Maa dialect of Mano (I will use Mano as a shortcut in the subsequent discussion) has four adnominal demonstratives used for exophoric reference: 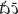, 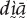, 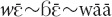 and *yā*∼*ā*∼*yāā*. Pronominal demonstratives are formed by adding 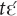 or *yé* (or only *yé*, in case of 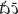) to the demonstrative stem: 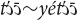, 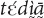, 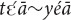 and 
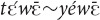
. They are assumed to be extensionally equivalent to the respective pronominal demonstratives and are occasionally mentioned in the paper (ex. 16). The demonstrative adverbs used in the language are: *zèē* “here,” 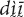 “there, distal” (which 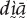 derives from), and *yī* “there, anaphoric.” They are not discussed in this article.

The demonstrative 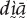 is obligatorily preceded by the marker of attention alignment 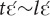. For the demonstratives 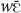 and *yā* the marker is optional. The demonstrative 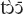 does not allow the marker of attention alignment, so combinations like 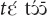 or 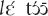 do not occur in the corpus and are disallowed in elicitation. This is most likely because it historically derives from a fusion between 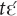 and the demonstrative 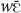
^3^. As is shown in the discussion of the semantics and pragmatics of these markers, the additional attention alignment effort is what distinguishes the typical contexts of use of the demonstratives 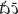 and 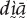, on the one hand, and 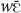 and *yā*, on the other.

Mano also has the demonstratives 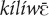 and *kílíā*, which are used exclusively for reference tracking. Given their limited scope and the fact that they are not used for exophoric reference, I will not discuss them further. In addition, Mano has a marker of bridging, *à*, which, in contrast with the demonstratives, is situated prenominally and is not part of the demonstrative paradigm (Khachaturyan, 2020b).

Table 1 presents the Mano demonstrative forms. The five forms (excluding the free variants) differ in the domain of use (exophoric only) for 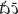 and 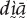; exophoric and endophoric (and simultaneously exophoric and endophoric) for 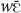 and *yā*; and endophoric only for 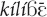 and *kílíā*. Further contrasts between the two pairs of forms, 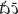 and 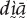, on the one hand, and 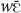 and *yā*, on the other, will be discussed in the following sections. The contrast between 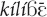 and *kílíā* should be an object of further investigation.

**TABLE 1:** Adnominal demonstratives in Mano.

Exophoric	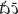
	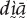
Exophoric/endophoric	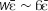
	*yā* ∼ *ā* ∼ *yāā*
Endophoric (anaphora)	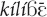
	*kílíā*

### Demonstratives 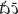 and 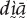


The demonstrative 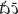 is used exclusively in the exophoric function to refer to objects present at the interactive scene; the act of reference is typically accompanied by a pointing gesture. In (3), the speaker is telling about his motorbike accident and is showing schematically on the ground where he was when the car hit him and how the car approached him.


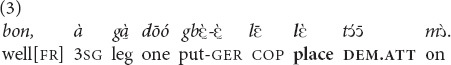


“[Here is the asphalted part of the road.] So one of its wheels is here (pointing to the ground, at the edge of the asphalted part). [He is behind me, he looks like he’s going to stop (at the side road)]” (MOC).

The demonstrative is speaker-anchored, which is evidenced by situations where the referent is invisible and inaccessible to the interlocutor, being relatively far away and in a different room (4).


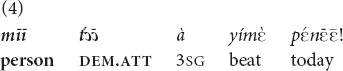


(Referring to his son who has just entered the room where he is sitting a man shouts outside to his wife:) “This guy (the boy), scold him today!” [fieldnotes].

The proximity of the referent to the speaker is flexible and not limited to the peripersonal space: while in (3), the object is within arm’s reach, in (4) it is about two meters away. My Mano interlocutors often introduce new people to me by pointing and saying 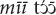 “this person” is such and such. The persons introduced can be at a considerable distance from me. What matters is that they are clearly visible and easily identifiable.

The demonstrative 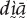 is used very rarely and I have only a few instances in my notes. It derives from a fusion of the deictic adverb 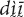 “there” with the marker *ā*^4^. Similarly to 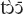, the demonstrative 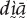 is used in the exophoric function, typically with a pointing gesture. While the preferred situation is where the object is visible, 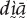 can occur with invisible objects that the speaker can locate with certainty (5, 6).


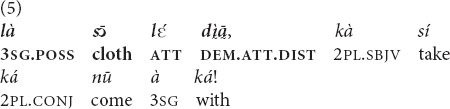


(The speaker is sitting on the floor, pointing to a basket on the opposite side of the room, about three meters away, asking her grandchild to bring her the clothes of another grandchild). “Those clothes of his, you (pl.) take them and bring them!” [fieldnotes].


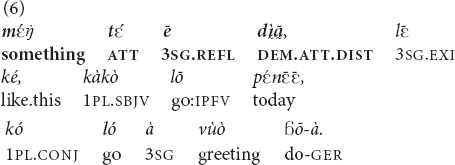


(An imaginary discussion between two inhabitants of the same village). “That guy over there (in the village, distance undetermined, may be visible, preferred interpretation, or invisible) is such (in such a state), let’s go today, let’s go and greet him” [MOC].

For both 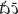 and 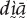 the distance between the speaker and the referent can vary. There is an overlap in the distance measures in 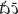 and 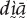: in (4) with 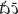 and (5) with 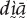 the distance is roughly the same. See also (7a), (7b) illustrating that both demonstratives are acceptable in certain contexts.


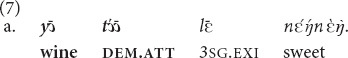


Situation A (The interlocutors sit one facing the other on opposite sides of the room, about 3 m away. The speaker is holding a can of beer in his hand). “This beer is good.”

Situation B (Same as above. The addressee is holding a can of beer in his hand. The speaker points to it and says). “This beer is good.”

Situation C (The interlocutors sit side by side. Either of them holds a can of beer in his hand). “This beer is good.”


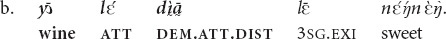


Situation A (The interlocutors sit one facing the other on opposite sides of the room, about 3 m away. The addressee is holding a can of beer in his hand). “That beer is good.”

Situation B (The interlocutors sit side by side, the beer is located on the other side of the room, about 3 m away). “That beer is good.”

^∗^The utterance is ungrammatical in a situation where the speaker is holding a can of beer in his hand [el.].

Just like 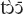, 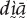 is a speaker-centered marker, since the location of the addressee does not affect its use (7a, 7b). In contrast with 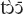, 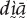 is never used to refer to objects in the peripersonal space (7b) and can be used with objects significantly further away from the speaker (6). Crucially, the spatial setting, the ongoing activity and the purpose of pointing may matter (although more examples are needed to confirm this). In (5), with 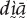, the object is located on the other side of the room. The speaker is sitting on the floor, the object is clearly out of reach and she is instructing her granddaughter to fetch the object—clothes—for her so that she could dress her newborn grandson. Thus, 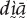 is used with objects which are not immediately accessible to the speaker by being outside of her engagement area defined as “the place which is, at moment t, the conceived site of a person’s currently dominant manual and attentional engagement” (Enfield, 2003, p. 89). In contrast, 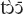 is neutral in that regard: it can be used with referents both within (3, 7a, Situation A, see also 26.1 below) and outside (4, 7b, Situation B) the engagement area.

### Demonstratives 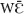 and yā

The demonstrative 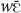, which also has the variants 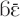 and *wāā*, and *yā*, which has the variants *ā* and *yāā*, can be used in all functions suggested by Himmelmann (1996) and Diessel (1999). They are very common in endophoric functions: referring to discourse itself (section “Discourse Reference”), reference tracking and anaphora (section “Anaphora”), or the recognitional function where the speaker assumes that the referent is identifiable for the interlocutor without prior mention (section “Recognitional Function”)^5^. Both demonstratives can also be used in the exophoric function, referring to objects present at the interactive scene (section “Exophoric Function: Indexing Familiarity”). They fulfill very similar functions to each other, and are very frequently interchangeable; I will gloss them as DEM1 and DEM2, respectively. I will begin with endophoric functions and then continue with the exophoric one, in which they contrast not only with each other, but also with the demonstratives 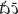 and 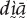 analyzed in the previous section.

#### Discourse Reference

The discourse referential function is not so frequently found in texts, but both demonstratives can be used in that function. The choice between them seems to be a matter of personal and/or dialectal preferences.


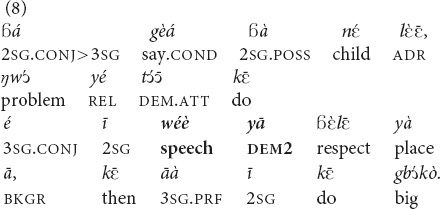


“If you say to your child: do this thing so that he pays respect to **that speech** of yours, (if he does so) then (it means that) he has honored you” [MOC].


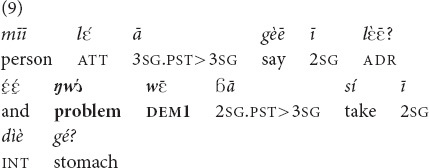


“[Pons Pilate said to Jesus: Man, is it you who are the king of Jews? And then he asked] Was it someone who told it to you? Or did you invent **that issue** (that you are the king of Jews) yourself (lit.: took **that problem** from your own stomach)?” [MOC].

#### Anaphora

In the anaphoric function, both demonstratives are widely used and seem to be interchangeable. *Yā* and 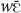 were attested in all speech genres available in our corpus, both monological ones (folktales, 10 and 11) or conversations (26).


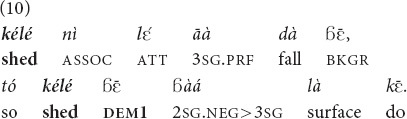


“Even the shed that has fallen, **that shed**, you don’t repair its roof” [MOC].

Example (11) is taken from a story about three hunters. The prior mention of the same referent with a 3pl pronoun occurred in the preceding clause.


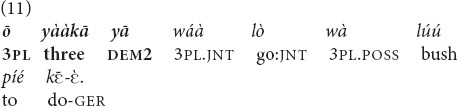


“[The story I want to tell concerns three hunters… Every month they go and hunt together very well]. **The three of them**, they went hunting” [MOC].

#### Recognitional Function

Both *yā* and 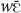 are particularly common in the recognitional function when they are used to refer to objects not present at the interactive scene, but are accessible via the common ground of the interlocutors (on that function in Mano, see Khachaturyan, 2019). Thus in example (12) both *tòò* “tomorrow” and 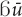 “rice” refer to entities made recognizable by a prior arrangement. “Everybody knows that I have to go tomorrow to my field to work,” the speaker told me when I asked her to comment on her usage of the demonstrative in (12).


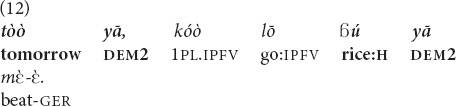


“**That tomorrow**, we will beat **that rice**” [fieldnotes].


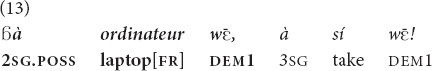


(An imagined conversation, where the speaker advises his addressee not to forget to take her laptop on the trip). “**This laptop of yours**, take it!” [el].

#### Exophoric Function: Indexing Familiarity

In the corpus 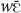 does not seem to show any clear preference. Example (2 above) was used with a referent close by, about 1.5 m away, while example (14) was used with a referent further away.


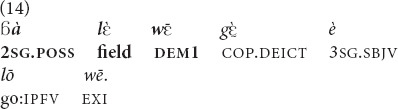


(An old man is showing, with a pointing gesture, to his daughter-in-law the placement and the direction of expanse of a field that he offered her and her husband). “Here is **this field of yours** (stretching to some 100 meters), it goes like this” [fieldnotes].

In (15), a woman instructs her brother-in-law to burn the feathers of a duck she is going to cook. The fireplace is some eight meters away from where she is sitting and is hidden behind a shed. I did not take a proper note of the position of her interlocutor.


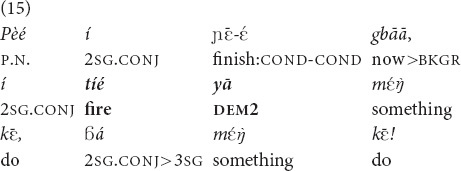


“Pe, when you have finished, do the thing with **that fire**, do the thing!” [fieldnotes].

In (16) the speaker is sitting at a table with his friend, eating dinner (with hands, as is customary among Mano). His wife approaches him from behind asking whether he has seen the charger to her phone, which she is holding in her hand. His response is given in (16). His hands are busy with eating, but he does not even need to point, he merely takes a quick look at her phone as he knows the model very well.


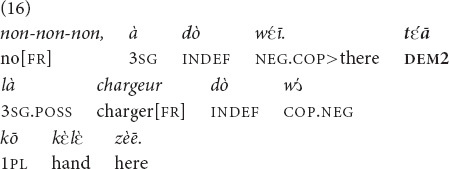


“[Do you(pl.) have a charger here?]” “No, we don’t. That (thing), we don’t have its charger here.”

The demonstratives 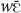 and *yā* may be used when either the speaker or both interlocutors do not see the referent. In (17), the speaker is riding on a motorcycle with the addressee and reminds his addressee to take the laptop, among other things, from a charging station, which is still out of sight.


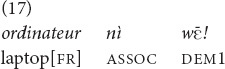


“The laptop (we are approaching the charging station)!” [fieldnotes].

Similarly, when I was discussing example (4) with my language consultant and asked what demonstrative form would be chosen if the boy were outside the house with his mother and the speaker inside, the consultant suggested the demonstrative *yā*, instead of 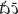 (18).


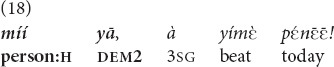


(A boy went into a puddle and came home dirty. He is outside the house with his mother, whom the father is addressing from inside the house). “That guy, scold him today!” [el].

Both 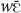 and *yā* can be used to attract the addressee’s attention to the referents which were not discussed in the prior discourse (2). And yet, in contrast with 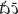 and 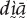, typically the speaker expects some existing familiarity with the referents, even when the demonstratives are used exophorically. In (15) the instruction of the speaker is very vague, “do the thing with the fire,” which means that they had already discussed the issue or that the addressee is used to those kinds of chores. In (14), there had clearly been some prior discussion of the field in the family. The old man is just showing his daughter-in-law where the field is before they start some bush clearing work. In (2), the speaker is a street vendor selling a very widespread Mano snack—popcorn balls. Prior familiarity with the object is what allows 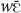 and *yā* to be used with invisible objects or objects to which it is difficult to draw the interlocutor’s attention if (s)he is busy with other things: such as riding a motorbike (2). Likewise, the speaker may be limited in her or his capacity to clearly point: because she is cooking (15) or eating (16), but pointing is typically not essential in reference retrieval. The demonstratives 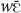 and *yā* are especially common in the anaphoric and recognitional functions, which rely on the cognitive accessibility of the referents alone without any clues from the physical context.

#### 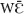 Vs. yā

In the real-life examples provided above, there is no clear tendency for the distance between the referent framed with the demonstratives 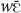 and *yā* and the deictic center. In elicitation, however, objects framed with 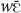 are presented as close to the speaker, while objects framed with *yā* are presented as further away. In (19a), repeated from 17, the speaker reminds his addressee to take the laptop, among other things, from a charging station. They were approaching it on a motorcycle and were already rather close, at the entrance to the town, so the speaker used 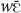. A contrasting example (19b), which would have been used had they been further away on the road, is with *yā.*


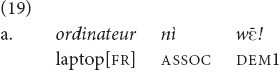


“The laptop (we are approaching the charging station!)” [fieldnotes].


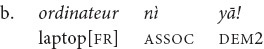


“The laptop! (don’t forget to pick it up when we pass by)” [el].

A similar contrast in the degree of familiarity may also affect the use of 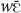 and *yā*. In (20), the demonstrative *wāā* (a variant of 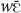) is used to refer to a woman that the addressee has just met for the first time at the local hospital, so she is highly salient in the context. In contrast, the woman’s husband, whom the addressee has never met but whose existence she may very well infer, given that the woman in question had just given birth to a child, is framed with the demonstrative *yā*. Both referents are out of sight and were not talked about in the prior conversation, but the woman is more familiar to the addressee than her husband. Note that here the speaker takes the addressee’s perspective in evaluating the referent’s relative familiarity.


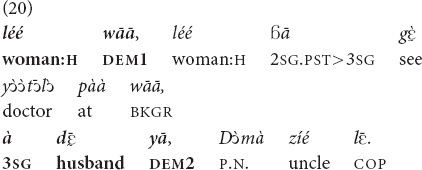


“That woman, that woman that you saw at the hospital, that husband of hers, he is Doma’s uncle” [fieldnotes].

In Mano, physical accessibility and engagement affects the use of 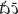 vs. 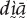. The difference between 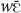 and *yā* is not yet clear from the data, but it is possible that a similar contrast is at play where engagement is seen in a more abstract way as a sphere of ownership, control, familiarity or mental preoccupation.

The objects referred to in (21) are expected to be served to the speaker by the addressee in the situation that the utterance describes. Therefore, although the referents are known to both parties in the interaction, which motivates the recognitional function, they belong to the sphere of the assumed control and possession of the speaker, so 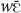 is chosen over *yā*.


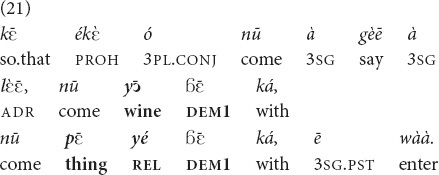


(A man’s mother and father died, but he did not have money to organize their funerals). “So that people don’t come saying: “Bring this wine, bring this thing” (the food and the drinks that invitees at a funeral are expected to be served), he ran away” [MOC].

Example (22) is from my notes of my consultant’s children commenting on pictures in a comic book. The children at that time were not fluent in French, so they could not read what was actually written in the word balloons and instead staged an imagined conversation between the book’s characters. The characters played with the referent (the ball) together and had equal access to the information in question.


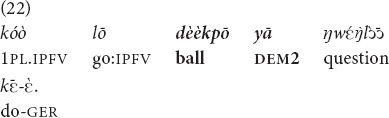


(Children were playing with a ball and accidentally threw it to the other side of the neighbor’s fence. They are deciding among themselves what to do with the ball). “We will ask about **that ball**” [fieldnotes].

Example (23) is taken from a conversation between relatives, two sisters-in-law, but it is the addressee who is more knowledgeable about the whereabouts of the referent, her children.


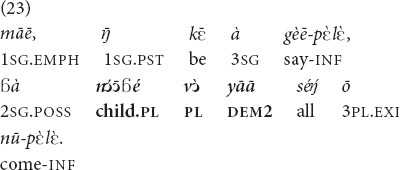


(A woman is talking to her sister-in-law, who came to celebrate the New Year with her, having brought only some of her children with her). “As for me, I thought that all of **those children** of yours were coming” [MOC].

Thus, the contrast between the demonstratives 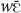 and *yā* implies the contrast in the engagement with the referent, where 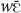 covers the engagement sphere of the speaker and *yā* is used for the common sphere or the addressee’s sphere. The contrast emerges from prior interactions and expectations that the interlocutors have: who owns and controls what (children, food served to a guest), what business is a matter of common concern, and what is taken as a personal matter.

### Semantics and Pragmatics of Mano Demonstratives

As argued above, the contrast within the two pairs of demonstratives, 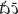 and 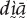, and 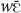 and *yā*, is motivated by the factor of the engagement sphere: 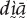 is used for referents outside the engagement sphere of the speaker and 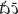 is neutral in that regard, while 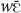 is used for referents within the engagement sphere of the speaker and *yā* is neutral. In the case of the former pair, engagement is understood in the sense of Enfield (2003) as an area of physical activity. In the case of the latter pair, engagement is seen in a more abstract way as an area of one’s expertise, familiarity or control (see Evans et al., 2018).

The referents of the noun phrase framed with 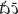 and 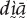 are objects that, as a rule, were not mentioned in the discussion immediately prior to the act of reference. Usually there is extra work needed to establish joint visual attention to the referent. The attention management marker 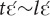 that is obligatorily used with 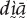 and that is likely embedded in the form of 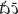 informs the addressee that she needs to align her attention with a non-trivial referent (Khachaturyan and Ozerov, in preparation). Gesture becomes a key means of securing joint attention and establishing reference and usually accompanies noun phrases with the demonstratives 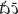 and 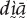.

In contrast, the common feature of all uses of 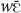 and *yā* listed above is that the referents are easily identifiable given the common ground of the interlocutors. And yet they are also compatible with deictic gesture, as shown in (14), and, more importantly, with the marker of attention alignment. Example (24) is taken from a spontaneous translation of Luke 9:35, where God announces that Jesus is his son and is chosen by him. Note that God is speaking from a cloud, which complicates reference resolution and triggers the use of the attention management marker. However, given the unusual circumstances of the referential act, no pointing is possible, so neither 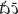 nor 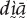 is possible in this context.


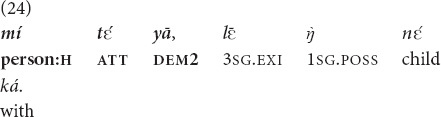


“**That person**, he is my Son” [MOC].

The attention management marker may be used to accompany 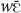 or *yā* when the referent is already in joint attention, but additional attention needs to be brought to it, as in emotional evaluations. Example (25) is taken from an explanation of the Bible episode where Jesus preaches in a synagogue in Nazareth, the town where he grew up. The Jews present in the synagogue know him well and are surprised that the “gracious” words are said by a man of such modest descent—the son of Joseph and Mary. Note that everyone is already attending to Jesus (“the eyes of everyone in the synagogue were fastened on him,” Luke 4:20, NIV). The attention management marker that the prayer leader employs in his explanation of the situation is used in the expression of surprise—similar to the emphatic use studied by Levinson (2004)—rather than to overcome the difficulty of attention alignment^6^.


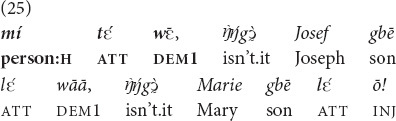


“That person, isn’t he JOSEPH’S SON, isn’t he MARY’S SON!” [MOC].

Thus, being-part-of-common-ground is not an invariant meaning of 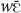 and *yā*, despite its frequent occurrence in natural demonstrative use. Instead, these demonstratives can be argued to have a general indicating function, DEM (Enfield, 2003). The use of a demonstrative in that function “presupposes that an addressee can know what it is referring to” (Enfield, 2003, p. 86). In contrast, 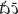 and 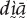, in addition to the indicating function, have the semantic function of drawing attention to a non-trivial referent. That 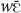 and *yā* are often used to indicate that the referent is part of the common ground is a pragmatic inference (Levinson, 2000): “the use of a semantically less specific or “weaker” form (given that a semantically more specific or “stronger” form is an option in the same grammatical context) implies the converse of the stronger form, yet without semantically encoding it” (Enfield, 2003, p. 86). In other words, because the speaker chooses not to use the semantically specific attention-drawing markers 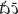 and 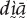, the addressee infers that extra work of attention alignment is not needed and that the referent is likely already available to her by virtue of the common ground she shares with the speaker. Yet, the inference can be overridden by an explicit use of the attention drawing marker 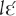. Table 2 summarizes the invariant semantics of the four Mano demonstratives from the least to the most specific.

**TABLE 2:** Semantics of Mano demonstratives.

*yā*	DEM
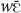	DEM, within speaker’s engagement sphere
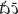	DEM, attention drawing
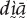	DEM, attention drawing, outside speaker’s engagement sphere

## Common Ground and Reference Resolution in Interaction

### Common Ground and Interactional History

A further layer of complexity arises when referential acts are seen not in isolation but as embedded in interactional sequences. As interaction unfolds within a given encounter and across encounters, more knowledge about referents, including those present at the interactive scene, becomes mutually available to the participants. The simplest case of mutual knowledge built in interaction and indexed by a demonstrative is anaphora. Indeed, the demonstratives *yā* and 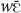, which are commonly used for reference tracking in monological texts (see section “Anaphora”), are also used in conversations for reference tracking across speech turns. In 26, the two interaction participants are engaged in cooking. In (26.1) the speaker A draws her addressee’s attention to the fish, which has a lot of bones. As joint attention is established, the speaker B uses *yāā* in the anaphoric function to confirm and elaborate on A’s observation (26.2).


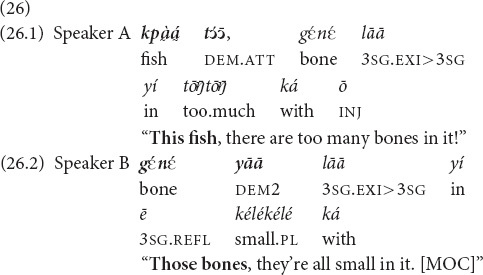


Example 26 is a “lean” case, where knowledge about referents is available from the interaction setting and discourse immediately preceding the use of the demonstratives. Section “Recognitional Function” presented further cases where some already available mutual knowledge was necessary for referent identification. The next three examples illustrate cases where referent identification is based on mutual knowledge which is assumed by the speaker but negotiated in interaction.

In (27), a woman (Speaker A) is helping her sister-in-law (Sister B) cook a festival dish, namely rice with mixed protein, fish and duck. Poulty is a more typical protein to be put in such a dish, so Speaker A is surprised they are adding fish and assumes that Speaker B is doing so to offer some food to her mother, who residing in a village called Gou and is known to be a fish lover. And yet it was another person who asked to prepare the fish. The person was first identified by Speaker B as “that woman” with the *ā* demonstrative (a variant of *yā*) in the recognitional function (27.4), and then Speaker A made sure they are talking about the same person by using a proper name, Maria (27.5).


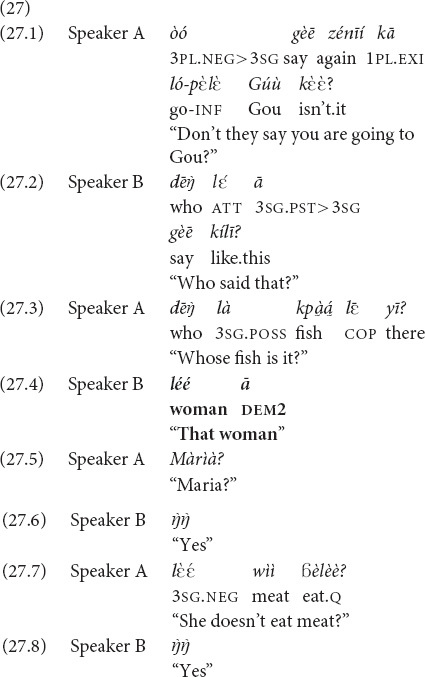


In addition to mutual knowledge, the engagement sphere factor structures local interactions, where a personal concern is put on the table and then taken up by the addressee as shared or, in contrast, a shared concern is projected and then recognized and validated by the addressee as hers. The next two examples illustrate that. In the elicited example (28) the speaker presents a referent as an object of his personal concerns and uses the demonstrative 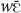, which encodes the speaker’s engagement area, while the addressee, ratifying the shared recognizability and at the same time conveying some additional information, uses the demonstrative *yā*.


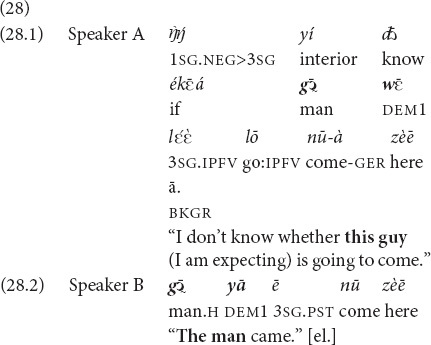


In (29), the sequence is inverse. The two speakers chat about several things, including a small eggplant plantation of one of their relatives, which keeps an elderly aunt of Speaker A busy (who is also the mother-in-law of Speaker B). By using the demonstrative *yā* and framing the issue as shared knowledge (29.1), A attempts to elicit a confirmation from her interlocutor that she follows what is being talked about. She receives feedback with the demonstrative 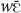, which indexes that the speaker recognizes the referent and includes it into her personal sphere (29.2).


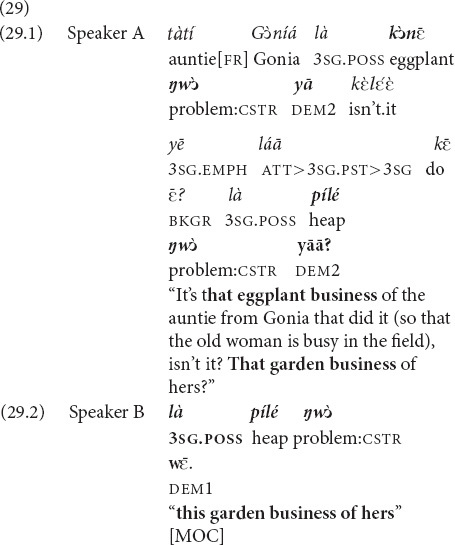


### Common Ground and Social Fields

The previous section illustrates that a typical referential act is embedded in an interactional sequence of reference resolution. At the same time it is embedded in the longer-term history of interaction between the given participants, allowing them to have access to mutual knowledge. Referential acts are also part of partially scripted social activities taking place in social fields where participants occupy particular positions with relations of power and reciprocity. As argued by Hanks, because of this embeddedness, the interactive space defined by deixis is “invested with much more specific values and relationships whose interpretation turns not on deixis,” but on a particular field (Hanks, 2005, p. 194). In particular, there is often domain-specific knowledge involved that the participants share even when a particular group of interactants has never communicated before.

The following example is an excerpt from a highly scripted type of discourse, a benediction ritual which is part of a traditional name-giving ceremony. The speaker, a classificatory nephew performing the benediction, utters a sequence of blessings to a newborn boy framed in the conjunctive verbal form (“let him be such,” “let such a thing happen to him”). The public responds by repeating the end of each token phrase of the benediction in the habitualis form (“he is such,” “such a thing happens to him”). The speaker refers to two abstract qualities (growing force, good intelligence) and one physical (a shining thing between the thighs, meaning well-functioning reproductive organs). Crucially, he refers to all three with the 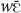 demonstrative because these are typical things to wish to a boy. The consistent use of the same demonstrative and the same tense forms endows the interaction with a rhythmical, routinized structure characteristic of the ritual context. At the same time, given that the boy is also present during the ritual, the reference to these qualities—especially the physical one—has an exophoric dimension.


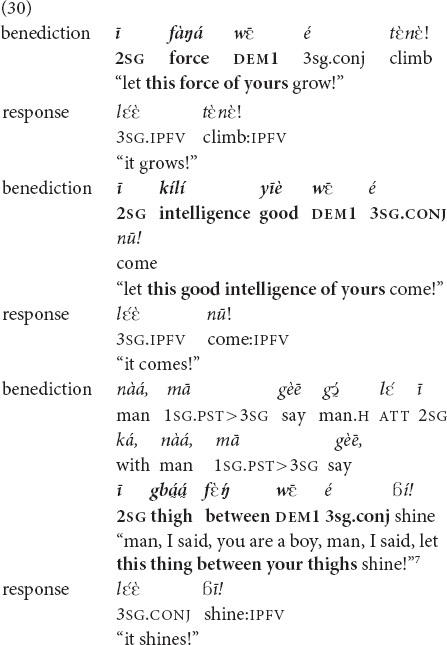


In some cases, the use of the demonstratives 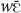 and *yā* does not only index the common ground and the routinized properties of interactions in a particular field, but also contributes to shape the context of interaction as a distinct social field with a presupposition of shared knowledge. Thus, in oral Bible translations as they are performed by Mano priests and prayer leaders, many noun phrases contain the demonstratives 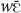 and *yā.* They are often used in first mentions of objects and places beyond the utterance context and perform the recognitional function. Many of these referents, however, are fairly exotic and cannot be assumed to be known by the community of Mano Catholics, such as the Horeb mountain in (31). Instead of indexing the shared knowledge of the referents in question, these deictic markers project it in a performative fashion. Because of the dialogic orientation of recognitional deixis, as a consequence of projection of recognizability, the speaker (a ritual specialist) and the addressees (the congregation) emerge as knowledge-sharing co-insiders. This, in turn, contributes to a performative creation of a community of co-insiders—a religious community sharing religious knowledge (Khachaturyan, 2019).


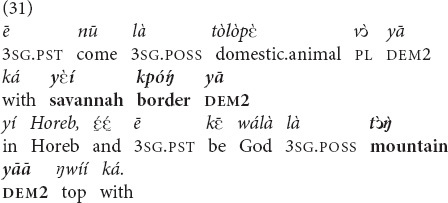


“He came with those domestic animals of his at **that border of the savannah**, at Horeb, that was a top of **that mountain** of God’s.”

French source: “Il mena le troupeau au-delà du désert et parvint à la montagne de Dieu, à l’Horeb.”

NIV: “… and he led the flock to the far side of the wilderness and came to Horeb, the mountain of God.”

Both (30) and (31) are examples of language use with an unusual participation framework where the speaker is a ritual specialist. Everyday family and village life is no less scripted than the fields of religious practice and is filled with routinized activities. It is the position of the wife of the elder brother that allows the speaker in (15) to give orders to her brother-in-law with minimal referential expressions (“do the thing with the fire”). Similarly, the seller of popcorn balls recurs to a recognizable marketing formula, “buy these X of yours” (2).

## Discussion

The relation of proximity is a function of the natural and social carving of the physical space. Such spatial divisions contribute to forming the notion of wider physical accessibility, which accounts for the use of speaker- or addressee-anchored forms (Burenhult, 2018). Furthermore, proximity is in certain cases a function of the bodily engagement of the interlocutors with the object and physical access to it. The notion of peripersonal space, which can be extended if the speaker uses tools (such as a stick) is in certain languages a better predictor of the choice of the demonstrative form than exact distance (Coventry et al., 2008).

As shown in examples (4) and (5) from Mano, however, the referent located at a similar distance outside the peripersonal space (about 2–3 m) may be framed with the marker 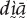 if the speaker intends to physically engage with the object but cannot reach it, or with the proximal marker 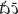 if mere pointing and identification is enough for the current purposes. If the speaker is busy with some chores and her hands are occupied, so that she cannot point (15) or if the addressee is busy with some tasks and cannot attend to pointing gestures (2), his attention can be called for by framing the referent as if it was invisible. Thus, the purpose of referent identification and the activity which it is part of motivate the choice of deictic marker. The engagement area, which is defined as “the place which is, at moment *t*, the conceived site of a person’s currently dominant manual and attentional engagement” (Enfield, 2003, p. 89) and which shifts depending on the interactional setting and the interlocutor’s current activity, matters for the choice of demonstrative reference sometimes more than physical distance *per se*. In Mano, the engagement area contrasts the demonstratives 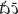 and 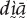, where the latter is situated outside the engagement area and the former is neutral in that regard. Engagement in a more abstract sense as an area of one’s ownership, control, or concern appears to contrast the markers *yā* and 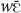, where the latter marks objects within the speaker’s engagement area and the former is neutral.

Attention focus management as an evolving interactive process which gets reflected by the choice of the demonstrative form has been recognized in much recent literature. A particularly well-known case is Turkish, mentioned in section “Introduction.” In Jahai, the addressee-centered marker *ton* is used when the addressee’s attention is already focused on the referent (Burenhult, 2003). Tiriyó is another language where the addressee’s attention focus, not physical distance, influences the choice between (two proximal) markers (Meira, 2018). In Mano, the demonstrative 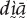 is always used with a marker of attention alignment 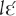 or 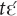. While the demonstrative 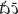 never combines with such a marker, it likely derives historically from a merger with 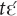. Rather than a means to express attention alignment across attempts at securing the addressee’s attention focus, the function common to both demonstratives is to underscore that the addressee needs to do some extra work to identify the referent; here, pointing at visible objects which often accompanies 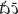 and 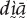 is a means to secure attention.

Demonstratives 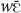 and *yā* can also be used with the attention alignment markers 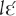 and 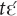 when there is some difficulty in referent identification (God speaking from a cloud and hence not being able to point) or there is some emphatic attention alignment. Thus, while 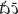 and 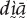 can be characterized as +ATTENTION ALIGNMENT, 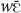 and *yā* are not -ATTENTION ALIGNMENT, but rather neutral and have the most general indicating function proper to demonstratives as a class (Enfield, 2003) as their primary meaning. Yet, in speech events they are often interpreted as marking referents that do not require additional attention alignment as they are already part of the common ground of the interlocutors. This interpretation arises from a pragmatic inference whereby the use of a semantically weaker term implies the opposite of the semantically stronger term that the speaker chose not to use. A similar contrast between a general indicating demonstrative and a demonstrative that indexes referents which require additional coordination between the speaker and the addressee is also attested in Yurakaré (Gipper, 2017).

The pragmatic function of the demonstratives 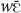 and *yā* of marking common ground in reference to objects present at the interactive scene is very frequently observed in interaction. Because these demonstratives do not have visual attention alignment as a necessary component of their semantics, 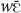 and *yā* are used in a variety of endophoric functions. Moreover, the markers 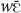 and *yā* grammaticalize into generalized clause-final markers used explicitly to mark backgrounded information. Thus, 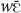 is also used in imperative clauses when the request or invitation is highly expected in the given context (see also the utterance final 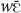 in 2 and 13); both 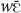 and *yā* are used as subordinate clause markers (see examples 8 and 10; Khachaturyan, 2018b).

The interlocutors’ common ground is difficult to assess in experimental settings and is much more rarely discussed as a parameter for the demonstrative choice. And yet it seems to be more basic than some other interactional parameters discussed in the literature. In particular, it often underlies the rationale for the choice of demonstrative for invisible referents and overrules the visibility parameter *per se*, as in Yélî Dnye (Levinson, 2005, 2018). Common ground seems to be a contrast for the “invisible” forms in Quileute (Andrade, 1933), where one set of markers denotes referents which are known to the addressee and another the referents which are known to both parties. It is because the referents are cognitively accessible that they are identifiable while being invisible. (In)visibility *per se* is not encoded in Mano but is a contextual factor that favors the use of the demonstratives 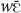 or *yā* that do not encode the attention coordination function and can be used to mark invisible, but cognitively available referents.

The way attentional and common ground distinctions are mapped into demonstrative systems varies from language to language. In Jahai, the same marker *ton* is used to confirm mutual attention to a referent, as well as in cases where referents form part of the common ground and for anaphoric reference (Burenhult, 2003). In Tiriyó, the marker used for referents already attended to can be used only for exophoric, and never for anaphoric reference (Meira, 2018). In Yélî Dnye, the common ground marker is used in the recognitional function, but not for anaphora, for which a dedicated marker exists (Levinson, 2018). In Yucatec Maya, the same non-immediate enclitic is used for recognitional and for anaphoric reference, as well as in exophoric uses where common ground is involved, but the contrast between the functions is expressed by a proclitic (Hanks, 2005). Finally, in Mano, the demonstratives 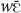 and *yā* cover all functions in the endophoric domain and are used for exophoric-cum-endophoric reference when common ground appears to matter.

Common ground arises from interactional history and broader cultural knowledge. On the one hand, any referential act in natural language use is part of an interactional sequence and interactional history involving the current interlocutors. On the other hand, it belongs to the domain of social action occurring in social fields that endow the interlocutors with social roles. This double embedding (in terms of Hanks, 2005) makes referents mutually known to the interlocutors, and therefore, cognitively available and anticipated. Thus, cognitive accessibility becomes one of the factors determining the choice of a deictic marker in exophoric reference. In Yucatec Maya, it is likely the shared interactional history, which is responsible for the routinized nature of certain types of interactions, such as greetings or scoldings, that triggers the choice of the non-immediate deictic over the immediate deictic in speaker-proximal settings (Hanks, 1990, 2005). In Mano, the demonstratives 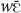 and *yā* are systematically used to mark referents in particular routinized speech genres, such as benedictions.

An additional complication regarding common ground is that it is not a fixed artifact: it can be creatively shaped by individuals and negotiated in interaction. Wrongly assuming common ground may lead to failures in recipient design (Deppermann, 2015) and additional interactional work in referent identification (Khachaturyan, 2019). Creative common ground management may become a feature of certain registers, as I show in the example of the Catholic register, where the use of demonstratives frames some referents as known to the congregation, while there are reasons to doubt their universal recognizability. This register feature arguably has broader consequences for shaping the interactional context, since it concomitantly shapes the addressees, the Catholic congregation, as a community of knowledge-sharing co-insiders.

## Conclusion

This paper is a first-hand ethnographic account of demonstrative reference in an under described language, Mano (Mande). It argues that in exophoric reference, the Mano demonstratives 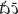 and 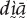 are contrasted with the demonstratives 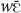 and *yā* in that the former index referents that require attention coordination for referent identification. In contrast, 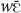 and *yā* are commonly interpreted, as a result of pragmatic inference, as the opposite of 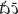 and 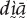 and index referents that do not require attention coordination being part of the common ground. They do not semantically encode common ground, however, having a more general referent identification function as their invariant semantics (Enfield, 2003; Gipper, 2017).

Common ground, including mutual knowledge of the referents, cannot be accounted for unless one studies demonstrative reference in natural use. Indeed, referential acts are part, first, of the immediate interactional sequence of referent identification and negotiation (Küntay and Özyürek, 2006), and second, in long-term interactional history involving given participants (Deppermann, 2018; Harjunpää et al., 2021). A further layer is the embeddedness of referential acts into the fabric of social action within particular social fields (Hanks, 2005). All these levels provide speech act participants with knowledge about referents which enable referent identification for objects both within and outside the interactive space. Thus, as argued by Agha (1996), Burenhult (2003), and most prominently Hanks (2005, 2011), cognitive access to referents functions alongside of perceptual access, which includes, but is not restricted to, spatial (visual) access. The centrality of common ground in reference to the objects at the interactive scene challenges the often assumed classification of demonstrative reference into exophoric and endophoric and, as a logical consequence of this, the primacy of exophoric reference at the level of the actual referential practice.

This article adds another argument in favor of viewing demonstrative reference as a social, interactive process (Peeters and Özyürek, 2016). It contributes to the empirical studies of (demonstrative) reference by bringing together the interactionist perspective and the sociological concept of field (Hanks, 2005) and by drawing on examples from distinct social domains of interaction, including everyday conversations and ritual discourse. The lack of a theoretical framework that would articulate the interlocking of interactional space, interactional history and social fields is a major shortcoming of this paper. A promising line of research which would support the development of such a framework would be a longitudinal study of (language) socialization within particular fields—how do people come to inhabit their social roles, know what they know and how is this process reflected in referential practice?

### List of Languages Cited and Their ISO 639-3 Codes

Jahai, Austroasiatic [jhi]; Mano, Mande [mev]; Quileute, Chimakuan [qui]; Tiriyó, Cariban [tri]; Turkish, Turkic [tur]; Yélî Dnye, isolate [yle]; Yucatec Maya, Mayan [yua]; Yurakaré, isolate [yuz].

## Data Availability Statement

The datasets for this article are not publicly available. The Mano Oral Corpus, on which some part of the research is based, will gradually be made publicly available at least in the anonymized and transcribed form and, current European GDPR allowing, also in the audio form. Some parts of the materials, namely, elicitation, can also be made available upon request. Fieldnotes, however, will not be made available since they contain sensitive information.

## Ethics Statement

Ethical review and approval were not required for the study on human participants in accordance with the local legislation and institutional requirements. Written informed consent for participation was not required for this study in accordance with the national legislation and the institutional requirements.

## Author Contributions

The author confirms being the sole contributor of this work and has approved it for publication.

## Conflict of Interest

The authors declare that the research was conducted in the absence of any commercial or financial relationships that could be construed as a potential conflict of interest.

## Footnotes

1 Diessel (1999) divides endophoric demonstrative use into four subfunctions: anaphora (*I saw a guy yesterday, that guy was clearly following me*), cataphora (*let me tell you this simple trick*), recognitional (as in Internet memes: *that feeling when*), and discourse deictic [*the moral of that story* (just been told)]. Levinson (2004) proposes a different classification, dividing demonstratives into two use-types, deictic (including exophoric, but also discourse deictic) and non-deictic, which includes recognitional, cataphoric and anaphoric uses plus the emphatic use (*that son of a bitch!*).

2 https://www.ethnologue.com/language/mev/23

3 Fusion is commonly present in Mano, especially in fast speech. However,  is used even in very slow and articulate speech; moreover, the fusion resulting in change of vowel quality () is not so frequently attested in Mano. For these reasons, I consider  to be a separate demonstrative marker, rather than a combination of  and .

4 Given that  is always used with a marker of attention alignment, which is also used to introduced relativized NPs (Khachaturyan and Ozerov, in preparation) and that *ā* is used to frame the right edge of relative clauses,  is interpretable both as an adnominal determiner “that X” and as part of a complex utterance of the type “X that is there.”

5 My corpus lacks examples of the cataphoric reference fulfilled with demonstratives.

6 The same attention management marker is also used to form focus constructions (see ex. 9, was it SOMEONE who told you, or you made it up?). On the relationship between attention management and focus, see Khachaturyan and Ozerov (in preparation).

## Abbreviations

ADR: addressee
ASSOC: associative plural
ATT: attention drawing
BKGR: backgrounding marker
COND: conditional
CONJ: conjunctive
COP: copula
DEM: demonstrative
DIST: distal
EMPH: emphatic
EXI: existential
FR: French
GER: gerund
H: high tone
INDEF: indefinite
INF: infinitive
INJ: interjection
INT: intensifier
IPFV: imperfective
JNT: conjoint
NEG: negative
PL: plural
POSS: possessive
PRF: perfect
PROH: prohibitive
PST: past
REFL: reflexive
REL: relativizer
SBJV: subjunctive
SG: singular.
